# Supplementary material for: Effect of ginger supplementation on the fecal microbiome in subjects with prior colorectal adenoma
Source: Sci Rep. 2024 Feb 5;14:2988. doi: 10.1038/s41598-024-52658-4 (PMC10844320; doi:10.1038/s41598-024-52658-4)
Supplement: Supplementary file 1 — Supplementary Information. [file 41598_2024_52658_MOESM1_ESM.docx]

**Supplemental Table S1. Cancer associated urinary biomolecule levels.** Urinary levels of creatinine, 11-dehydro-thromboxane B2, and prostaglandin E2


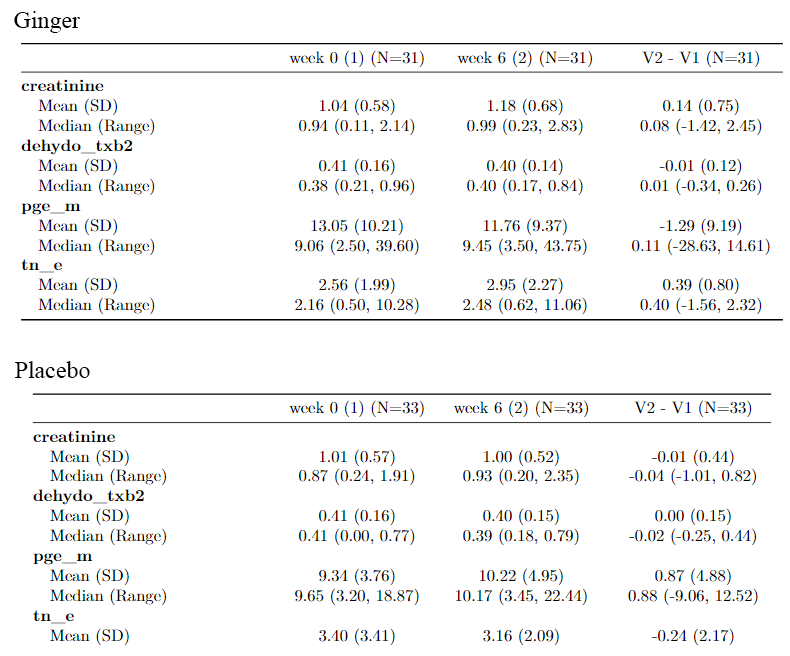


**Supplemental Figure S2. Dietary and medication intake survey.
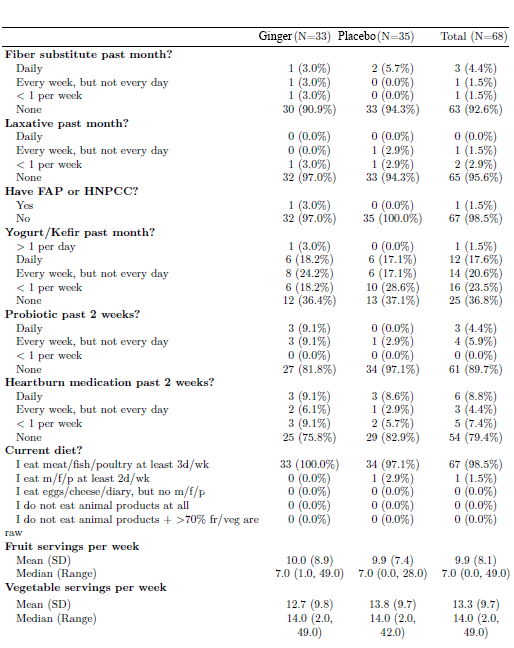
**

**Supplemental Figure S3. Stool Kit Component List**

Fisherbrand Commode Specimen Collection System

Monosol Water soluble bag 12" x 18" 1.5 mil

Sarstedt - Tube, Collecting/Mailing Feces Samples 76 x 20mm

Sarstedt - Mailing Container 30x85mm 40ml PP

Walter Stern Economical Solid Glass Beads for Distillation Columns; Approx. dia. 3mm

Chemicals Ethyl Alcohol, Anhydrous (Ethanol) 200 Proof

HealthLink Deionized Water Reagent Grade

Polar Tech, insulated shipping boxes 6 x 4-1/2 x 3

Sonoco ThermoSafe U-tek Phase Change Materials - freezer packs

Bag, Reclosable Biohazard; Minigrip LLC; SPECI-ZIP; Transparent, printed biohazard warning; Bilingual in English and Spanish; Designed with info pocket (3 wall), tear line; Size: 6 x 9 in.

Therapak Absorbent Materials 3 x 4 in. sheets
